# LPS Cooperates with Poly-L-Arginine to Promote IL-6 and IL-8 Release via the JNK Signaling Pathway in NCI-H292 Cells

**DOI:** 10.1155/2016/3421060

**Published:** 2016-12-26

**Authors:** Ling-Ling Zhang, Bing Chen, Xiao-Yun Fan, Sha-Sha Wu, Sheng-Quan Zhang, Hui-Mei Wu

**Affiliations:** ^1^Department of Geriatric Respiratory Medicine, The First Affiliated Hospital of Anhui Medical University, Hefei 230022, China; ^2^Department of Biochemistry and Molecular Biology, Anhui Medical University, Number 81, Meishan Road, Hefei, Anhui 230022, China

## Abstract

*Objective*. Herein, we aimed to study the mechanism whereby poly-L-arginine (PLA) and lipopolysaccharide (LPS) can synergistically induce the release of interleukin-6 (IL-6) and IL-8 in NCI-H292 cells.* Methods*. NCI-H292 cells were divided into control, PLA, LPS, and PLA+LPS groups. At various time points, the phosphorylation of JNK in each group was measured by western blotting. Additionally, the productions of IL-6 and IL-8 were assessed using an enzyme-linked immunosorbent assay (ELISA). The effects of SP600125, an inhibitor of the JNK pathway, on the increase of p-JNK, IL-6, and IL-8 were also studied.* Results*. Our results showed that either PLA or LPS treatment alone can significantly increase the phosphorylation level of JNK in NCI-H292 cells. Of interest was the combined use of PLA and LPS that has a synergistic effect on the phosphorylation of JNK, as well as synergistically inducing the release of IL-6 and IL-8 in NCI-H292 cells. Furthermore, SP600125 significantly inhibited the activation of JNK signal, as well as reducing the productions of IL-6 and IL-8 in response to PLA+LPS stimulation.* Conclusions*. The JNK signaling pathway contributes to the release of IL-6 and IL-8, which is stimulated by the synergistic actions of PLA+LPS in NCI-H292 cells.

## 1. Introduction

Bronchial asthma is a pulmonary disease that is characterized by recurrent episodes of reversible airway stenosis, airway hyperresponsiveness (AHR), and chronic airway inflammation, which is associated with various cells and cellular components [1].

Airway epithelial cells, which can release proinflammatory cytokines, such as IL-6 and IL-8, via autocrine and paracrine mechanisms [2, 3], may accelerate the development and progression of asthma under the actions of both immunologic and nonimmunologic stimuli [4, 5]. Moreover, cytokines can promote the migration of inflammatory cells and aggravate airway inflammation in asthmatic patients.

Eosinophils are a key effector cell type that play an important role in the pathogenesis of asthma [6]. The presence of eosinophils in the airway lumen and lung tissues is often regarded as a defining feature of asthma [7]. The infiltration of eosinophils has been reported in the peripheral blood, bronchial tissues, and bronchoalveolar lavage fluid (BALF) of asthmatics, and a correlation between the number of eosinophils and extent of damage in the airway epithelium has been established [8]. The role of eosinophils in asthma is a consequence of their abilities to secrete eosinophil granule proteins, including major basic protein (MBP), which is highly basic and cationically charged [9]. MBP released from activated eosinophils may generate AHR by either direct effects on airway myocytes or indirect effects [10] through the release of epithelial-derived mediators [11–13], resulting in increased epithelial permeability [14], cytotoxicity [15], or the inhibition of muscarinic M2 receptors [16]. The effects of MBP can be mimicked by PLA, a synthetic cationic polypeptide. PLA can damage cell membrane structure and function of the airway epithelium by creating many pores, resulting in a reduction in the number of desmosomes and the induction of an inflammatory pathology [17].

LPS, an endotoxin derived from Gram-negative bacteria, can be found ubiquitously in our environment in places such as milk, tobacco smoke, textiles, and particulate air pollution. Recent studies have suggested that inhaled endotoxin plays a vital role in the pathogenesis of asthma. A conspicuous increase in inflammatory cell counts of eosinophils and neutrophils, as well as the levels of IL-6, IL-8, and eosinophil cationic protein, was observed following LPS plus dust mite challenge [18]. LPS coadministered with ovalbumin prolonged responses to histamine and induced increased numbers of inflammatory cells in guinea pigs [19]. These findings suggested that LPS exposure may exacerbate airway inflammation in asthma.

MAPK signaling pathways are critical to many cellular processes, and their activation induces inflammatory cell factor and chemokine production in bronchial epithelial cells [20]. The c-Jun NH2 terminal kinase (JNK) is one of three MAPK subfamilies, among which the JNK pathway is associated with chronic inflammation. As Wang et al. showed, LPS can induce the inflammation of bronchial epithelial cells via JNK signaling [21] and induced the release of the cytokines IL-6 and IL-8 [22, 23]. Our previous study has established that PLA acted synergistically with LPS to promote the release of IL-6 and IL-8 via the P38/ERK signaling pathway in NCI-H292 cells [24]. Therefore, we hypothesized that intracellular JNK pathways may be also involved in the LPS-PLA-induced release of proinflammatory factors by airway epithelial cells. As the phosphorylation of JNK was blocked, expression levels of IL-6 and IL-8 were also either reduced or absent, suggesting a potential role for JNK signaling pathways in the LPS-PLA-induced release of proinflammatory factors by human airway epithelial cells.

## 2. Materials and Methods

### 2.1. Reagents

LPS, PLA, and SP600125 were purchased from Sigma-Aldrich (St. Louis, MO, USA). Dimethyl sulfoxide, which was also purchased from Sigma-Aldrich, was used as a solvent for SP600125. Fetal bovine serum was purchased from Life Technologies Corporation (Carlsbad, CA, USA). Anti-JNK was purchased from Zhong Shan-Golden Bridge (Beijing, China). Anti-phospho-JNK and anti-*β*-actin were purchased from Santa Cruz Biotechnology (Dallas, TX, USA). Chemiluminescence reagents were purchased from Thermo Scientific (Rockford, IL, USA). ELISA kits for the detection of IL-6 and IL-8 were purchased from Senxiong (Shanghai, China).

### 2.2. Cell Culture

NCI-H292 cell lines and lymph node metastases of a pulmonary mucosa epithelial cell carcinoma were purchased from the cell bank of the Type Culture Collection of the Chinese Academy of Science (Shanghai, China). NCI-H292 cells were cultured and propagated in RPMI-1640 medium (Thermo Fisher Scientific) supplemented with 10% fetal bovine serum (Life Technologies) in a humidified atmosphere of 5% CO_2_/95% air at 37°C. The culture medium was changed every 48–72 h. Optimal cellular behavior was obtained at 70%–80% confluence, and when cells reached this density, they were digested with trypsin, centrifuged, counted, and seeded into 24-well culture plates at a density of 3 × 10^5^ cells/well and then cultured. At 80% confluence, 500 *μ*L per well serum-free RPMI-1640 medium was added to maintain the cells for 24 h for mediator quantification. Previously, our group has shown that the optimal concentration of LPS or PLA for inducing airway epithelia cell inflammation was 5 *μ*g/mL. NCI-H292 cells were exposed to 5 *μ*g/mL LPS or/and 5 *μ*g/mL PLA for the indicated periods of time. Cells were incubated with signaling pathway inhibitors prior to LPS or/and PLA treatment under the specified conditions.

### 2.3. Western Blotting

NCI-H292 cells were divided into groups of cells treated with LPS, PLA, or PLA+LPS; specifically, each group was treated with 5 *μ*g/mL LPS or/and 5 *μ*g/mL PLA for 0, 15, 30, 45, 60, 90, or 120 min. In other experiments, NCI-H292 cells were divided into control, PLA, LPS, PLA+LPS, PLA+SP600125, LPS+SP600125, PLA+LPS+SP600125, and SP600125 groups. Before stimulation with 5 *μ*g/mL LPS or/and 5 *μ*g/mL PLA for 60 min, cells were preincubated for 2 h with SP600125 (30 *μ*M), a p-JNK signaling pathway inhibitor.

After incubations, cells were washed with cold phosphate-buffered saline (PBS) 2-3 times, and whole cell protein lysates were extracted in RIPA buffer (0.1% SDS, 1% Nonider-P40, 150 mM NaCl, 0.5% deoxycholic acid, and 50 mM Tris-HCl [pH 7.4]) that contained the protease inhibitor phenylmethanesulfonyl fluoride (PMSF). Total protein extracts were separated by 15% SDS–PAGE and transferred onto a polyvinylidene fluoride (PVDF) membrane. Each membrane was blocked with 5% nonfat milk in Tris-buffered saline with Tween 20 (TBS-T) at room temperature for 2 h, then was washed with TBS-T every 10 min three times, and incubated overnight with respective primary antibodies at appropriate dilutions (1 : 250 for rabbit anti-human JNK or mouse anti-human p-JNK antibodies or 1 : 500 for mouse anti-human *β*-actin antibody) at 4°C. Then, membranes were washed with TBS-T, treated with corresponding IgG-HRP conjugated secondary antibody (1 : 6000 for goat anti-rabbit secondary antibody for rabbit anti-human JNK antibody, 1 : 2000 for goat anti-mouse secondary antibody for mouse anti-human p-JNK antibody, or 1 : 10,000 for goat anti-mouse secondary antibody for mouse anti-human *β*-actin antibody) at room temperature for 2 h, washed with TBS-T, and incubated with general chemiluminescence reagents (ECL, Thermo Scientific) to detect the respective protein bands. Each experiment was repeated at least three times.

### 2.4. Enzyme-Linked Immunosorbent Assay (ELISA)

NCI-H292 cells in 24-well culture plates were washed with RPMI-1640 medium without fetal calf serum. Cells were divided into control, PLA (5 *μ*g/mL), LPS (5 *μ*g/mL), PLA (5 *μ*g/mL) + LPS (5 *μ*g/mL), PLA (5 *μ*g/mL) + SP600125 (30 *μ*M), LPS (5 *μ*g/mL) + SP600125 (30 *μ*M), PLA (5 *μ*g/mL) + LPS (5 *μ*g/mL) + SP600125 (30 *μ*M), and SP600125 (30 *μ*M) groups. Prior to stimulation with LPS or/and PLA for 60 min in RPMI-1640 medium that lacked fetal calf serum for 24 h, NCI-H292 cells were incubated with SP600125 (30 *μ*M) for 2 h. Then, cell culture supernatants were centrifuged and collected. ELISA commercial kits were used to detect the levels of IL-6 and IL-8 in samples according to the manufacturer's instructions. All assays were completed in duplicate and mean values were calculated.

### 2.5. Data Analysis

Statistical analyses were performed using SPSS version 17.0 (SPSS Inc., Chicago, IL, USA). All values were displayed as means ± standard error of the mean. One-way Analysis of Variance (ANOVA) was applied for comparisons of more than two groups, and LSD was used when equal variance was assumed between groups or Dunnett's* T*_3_ was used when no equal variance was observed. *P* values less than 0.05 were considered to denote statistically significant differences.

## 3. Result

### 3.1. PLA and LPS Activate the JNK Signaling Pathway in NCI-H292 Cells

**Figure 1:**
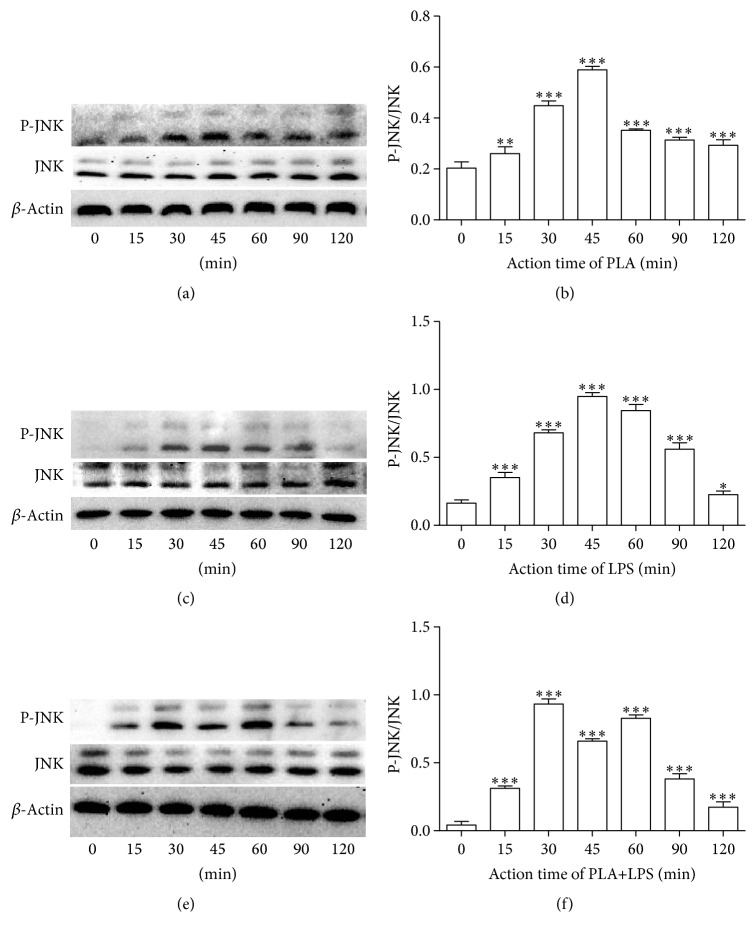
Activation of the JNK signaling pathway by PLA or LPS in NCI-H292 cells. The JNK signaling pathway is activated by 5 *μ*g/mL PLA, 5 *μ*g/mL LPS, or 5 *μ*g/mL PLA + 5 *μ*g/mL LPS in NCI-H292 cells. Western blot analysis showed expression of JNK and the phosphorylated form of JNK (p-JNK) at various time points (0, 15, 30, 45, 60, 90, and 120 min). *β*-actin was used as an internal protein loading control. ((a) and (b)) The expression level of phosphorylated JNK in NCI-H292 exposed to PLA was increased. ((c) and (d)) The expression level of phosphorylated JNK in NCI-H292 exposed to LPS was increased. ((e) and (f)) The expression level of phosphorylated JNK in NCI-H292 exposed to PLA+LPS was increased prominently. Statistical analyses were compared with the beginning time point: ^*∗*^*P* < 0.05, ^*∗∗*^*P* < 0.01, and ^*∗∗∗*^*P* < 0.001.

To test whether PLA or LPS could activate the JNK signaling pathway in NCI-H292 cells, protein expression levels of p-JNK and JNK were measured by western blotting. The protein bands on blots were normalized to the corresponding *β*-actin band. The expression level of phosphorylated JNK in NCI-H292 exposed to 5 *μ*g/mL PLA for 15, 30, 45, 60, 90, and 120 min was increased compared with that of the control group, most prominently at 45 min (*P* < 0.01, Figures 1(a) and 1(b)). A similar result was found in NCI-H292 cells stimulated with 5 *μ*g/mL LPS (*P* < 0.05, Figures 1(c) and 1(d)) or 5 *μ*g/mL PLA + 5 *μ*g/mL LPS (*P* < 0.001, Figures 1(e) and 1(f)). Notably, the ratio of p-JNK to JNK in NCI-H292 cells exposed to PLA+LPS for 30 min was greater than that of other time points.

### 3.2. PLA+LPS Synergistically Activates the JNK Signaling Pathway

**Figure 2:**
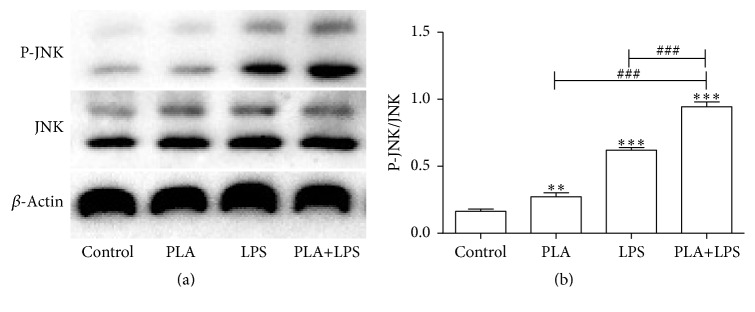
Synergistic effect of PLA+LPS on JNK signaling in NCI-H292 cells. The expression levels of p-JNK and JNK were measured by western blotting in NCI-H292 cells stimulated with 5 *μ*g/mL PLA, 5 *μ*g/mL LPS, or 5 *μ*g/mL PLA + 5 *μ*g/mL LPS for 60 min; *β*-actin was used as an internal control. The PLA+LPS group showed a further increased level of p-JNK, which was greater than that of LPS or PLA alone. Significant differences are shown as follows: compared with the control group, ^*∗∗*^*P* < 0.01, ^*∗∗∗*^*P* < 0.001; compared with PLA+LPS group, ^###^*P* < 0.001.

NCI-H292 cells were incubated with 5 *μ*g/mL PLA, 5 *μ*g/mL LPS, or 5 *μ*g/mL PLA + 5 *μ*g/mL LPS for 60 min. We detected a significant increase of p-JNK as that of compared with the control group (*P* < 0.01). The PLA+LPS group showed a further increased level of p-JNK, which was greater than that of LPS or PLA alone (*P* < 0.001; Figure 2).

### 3.3. Synergistic Effects of PLA+LPS Are Mediated by Activation of the JNK Signaling Pathway

**Figure 3:**
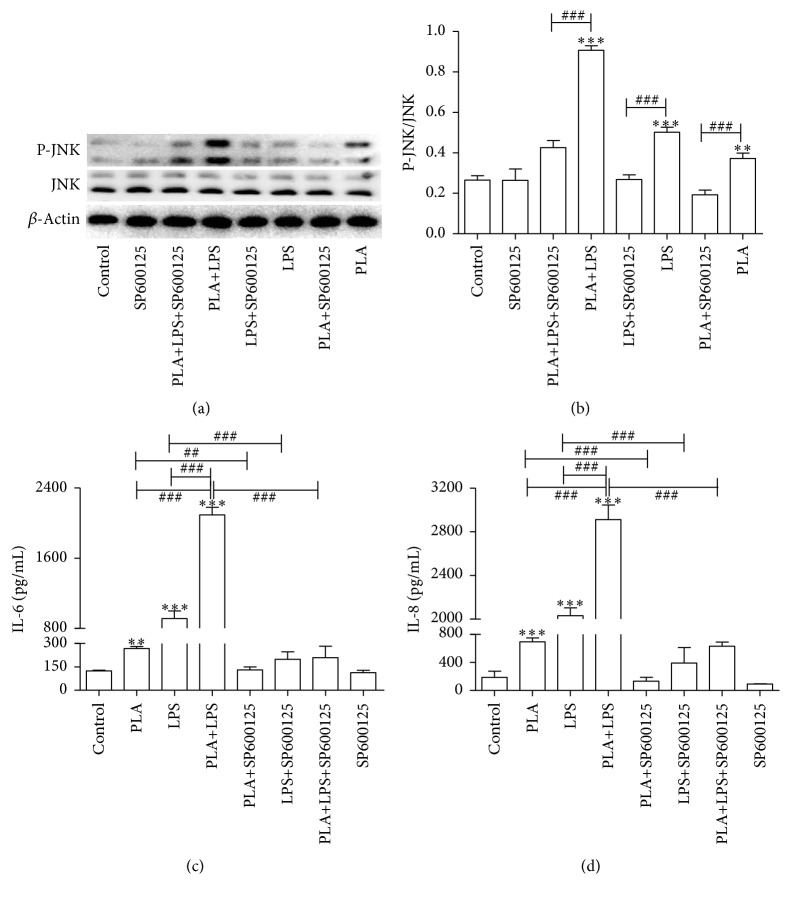
The JNK pathway inhibitor SP600125 alters the release of IL-6 and IL-8 by NCI-H292 cells. NCI-H292 cells were stimulated with 5 *μ*g/mL PLA, 5 *μ*g/mL LPS, or 5 *μ*g/mL PLA + 5 *μ*g/mL LPS for 60 min after preincubation with SP600125. The expressions of p-JNK and JNK were measured using western blotting. *β*-actin was used as an internal control. Production of IL-6 and IL-8 was measured by ELISA. ((a) and (b)) The expression level of phosphorylated JNK in NCI-H292 exposed to PLA, LPS, or PLA+LPS was inhibited effectively by the inhibitor SP600125. ((c) and (d)) PLA+LPS stimulated significantly more IL-6 and IL-8 production than either PLA or LPS treatment alone. The synergistic effects of PLA+LPS on the release of IL-6 and IL-8 could be blocked by SP600125. Significant differences are shown as follows: compared with the control group, ^*∗∗*^*P* < 0.01, ^*∗∗∗*^*P* < 0.001; compared with the PLA+LPS group, ^###^*P* < 0.001; PLA compared with PLA+SP600125, LPS compared with LPS+SP600125, and PLA+LPS compared with PLA+LPS+SP600125, ^###^*P* < 0.001.

Increased levels of p-JNK in NCI-H292 cells stimulated with PLA+LPS, PLA, or LPS for 60 min were blocked by SP600125, an inhibitor of the JNK signaling pathway (*P* < 0.001, Figures 3(a) and 3(b)).We measured the levels of IL-6 (Figure 3(c)) and IL-8 (Figure 3(d)) stimulated by PLA+LPS, or either PLA or LPS for 60 min in supernatants by ELISA. Both PLA and LPS alone induced the releases of IL-6 (PLA, *P* < 0.01; LPS, *P* < 0.001) and IL-8 (*P* < 0.001) contrast with the control cells. PLA+LPS stimulated significantly more IL-6 and IL-8 production than either PLA or LPS treatment alone (*P* < 0.001). After preincubated SP600125 (30 *µ*M) for 2 h, the NCI-H292 cells released less IL-6 (PLA, *P* < 0.01; LPS and PLA+LPS, *P* < 0.001) and IL-8 (*P* < 0.001) when stimulated by PLA+LPS, or either PLA or LPS alone. Thus, the synergistic effects of PLA+LPS on the release of IL-6 and IL-8 could be blocked by the inhibition of the JNK signaling pathway.

## 4. Discussion

Previously, we have shown that LPS can exaggerate PLA-induced IL-6 and IL-8 production by airway epithelial cells via an uncharacterized mechanism [25]. This present study aimed to assess whether the JNK signal transduction pathway is involved in enhanced IL-6 and IL-8 production by NCI-H292 cells stimulated with PLA+LPS.

Allergic bronchial asthma is a chronic inflammatory disorder of the airways in which various cells and cellular components act. This disease entity represents a heterogeneous group of different airway inflammation patterns that show different latent underlying immune mechanisms [26]. Many inflammatory mediators have been shown to regulate the maintenance and chronicity of inflammatory reaction through the secretion of cell factors from epithelial, endothelial, and constitutive mesenchymal and immune cells [27]. The airway epithelium is a key contributor to the pathogenesis of asthma and has been shown to generate excess inflammatory and proinflammatory mediators, such as IL-6 and IL-8 [28]. Neveu and colleagues also established that airway epithelial cells are the main producers of IL-6 in the lung upon exposure to allergens in vivo [29]. IL-6 is generally considered to be a nonspecific inflammatory marker and an active modulator of the immune response that can inhibit Th1 cell differentiation and exacerbate the imbalance between Th1 and Th2 cells [30]. Overexpression of IL-8 causes both neutrophil recruitment and chemotaxis, which is a sign of virus-induced asthma exacerbation [31, 32]. Both IL-6 and IL-8 can exacerbate asthmatic inflammation.

LPS is known to exert its effects by binding to the TLR4 receptor on the surface of airway epithelial cells [33] to induce the transcription and translation of various proinflammatory cytokines and chemokines, including IL-6 and IL-8 [34, 35]. After the exposure of NCI-H292 cells to PLA+LPS, there was a marked increase of IL-6 and IL-8 compared with cells treated with PLA or LPS alone, indicating there was a synergistic effect of PLA+LPS on the expression of IL-6 and IL-8.

Other studies have shown that the JNK pathway has crucial roles in the regulation of LPS-induced inflammation [22]. After exposure to PLA, LPS, or PLA+LPS for various amounts of time, the activation of JNK could be detected in NCI-H292 cells. Our western blot results showed the JNK signal transduction pathway was activated in NCI-H292 cells with maximal expression of P-JNK/JNK at 45 min when induced by PLA or LPS, and at 30 min when induced by PLA+LPS. Thus, we confirmed that the JNK signal transduction pathway was activated in NCI-H292 cells by PLA, LPS, or PLA+LPS. Importantly, the synergistic effect of PLA+LPS on the activation of JNK signaling was demonstrated by measurements of p-JNK in NCI-H292 cells stimulated by PLA, LPS, PLA+LPS for the same amount of time (60 min). Notably, p-JNK expression was most pronounced upon stimulation with PLA+LPS compared with PLA or LPS alone.

Using genetic inhibitors, Holtmann et al. have shown that JNK activation regulates IL-8 production at the transcriptional and translational levels in human embryonic kidney cells [36]. Additionally, in a human airway epithelial cell line, JNK regulated IL-8 promoter activity in a NF-*κ*B-dependent manner [22]. Binding of LPS to its cognate receptors on immune cell membranes, such as in RAW 264.7 macrophage cells, activated several signaling pathways, including the JNK signaling [37] cascade that promotes the expression of proinflammatory cytokines, including IL-6 [38]. These data demonstrated that the JNK pathway is vital to the LPS-induced generation of IL-6 and IL-8. When SP600125, a specific JNK inhibitor, was used to preincubate with NCI-H292 cells, we found that the expression levels of IL-6 and IL-8 induced by PLA, LPS, or PLA+LPS displayed an apparent reduction. The specific JNK inhibitor prevented the increased release of IL-6 and IL-8 that was induced by PLA+LPS. Therefore, we conclude that JNK signaling contributes to the increased expression levels of IL-6 and IL-8 induced by PLA, LPS, PLA+LPS.

In conclusion, we provide evidence that LPS acts synergistically with PLA to activate the release of the proinflammatory cytokines IL-6 and IL-8 in NCI-H292 cells, and JNK signaling contributes to this process. However, according to the existing studies [39], the JNK inhibitor SP600125 also acts as an antagonist of the aryl hydrocarbon receptor which played a role in modulation of inflammatory signals, including altered expression of proinflammatory cytokines and deregulated expression of principle enzymes producing inflammatory mediators [40]. This effect may be synergistic with JNK signaling pathway comediating release of the proinflammatory cytokines IL-6 and IL-8. Therefore, further studies are needed to fully elucidate the molecular mechanism for increased IL-6 and IL-8 production.
